# Immune Responses in Parkinson's Disease: Interplay between Central and Peripheral Immune Systems

**DOI:** 10.1155/2014/275178

**Published:** 2014-04-13

**Authors:** Xiaomin Su, Howard J. Federoff

**Affiliations:** ^1^Department of Neuroscience, Georgetown University Medical Center, Washington, DC 20057, USA; ^2^Department of Neurology, Georgetown University Medical Center, Washington, DC 20057, USA

## Abstract

The etiology of Parkinson's disease (PD) is complex and most likely involves numerous environmental and heritable risk factors. Recent studies establish that central and peripheral inflammation occurs in the prodromal stage of the disease and sustains disease progression. Aging, heritable risk factors, or environmental exposures may contribute to the initiation of central or peripheral inflammation. One emerging hypothesis is that inflammation plays a critical role in PD neuropathology. Increasing evidence suggest that activation of the peripheral immune system exacerbates the discordant central inflammatory response and synergistically drives neurodegeneration. We provide an overview of current knowledge on the temporal profile of central and peripheral immune responses in PD and discuss the potential synergistic effects of the central and peripheral inflammation in disease development. The understanding of the nature of the chronic inflammation in disease progression and the possible risk factors that contribute to altered central and peripheral immune responses will offer mechanistic insights into PD etiology and pathology and benefit the development of effective tailored therapeutics for human PD.

## 1. Introduction

Parkinson's disease (PD) is a progressive, age-related, neurodegenerative disorder. Clinically, it is characterized by motor symptoms, such as rigidity, bradykinesia, postural instability, gait disorder, and tremor [1]. However, nonmotor symptoms, such as hyposmia, gastrointestinal abnormalities, and autonomic dysfunction are increasingly accepted as integral parts of PD clinical manifestations and often precede the classical motor symptoms [2]. Pathologically, selective neurodegeneration in the nigrostriatal circuit, presence of dysregulated immune activation, and the occurrence of Lewy bodies (LB) in central and peripheral nervous systems are observed in PD [3]. The cause of nigral neurodegeneration in PD and its underlying mechanisms remain elusive; however, involvement of inflammatory events has been postulated to contribute to neuronal loss. Indeed, inflammation has been linked to many other age-related chronic neurodegenerative diseases, including Alzheimer's disease [4], amyotrophic lateral sclerosis [5, 6] and Huntington's disease [7, 8]. Triggering factors of inflammation may be dysregulation of inflammatory pathways (e.g., immune alteration associated with aging or genetic vulnerability), pathogens (e.g., bacterial or viral infection), environmental toxins (e.g., pesticides), and protein aggregates (e.g., *α*-synuclein (*α*-Syn)).

There has been considerable debate in the field as to whether inflammation is a driving force in neurodegeneration or simply represents a response to neuronal death. Here, we describe the temporal profile of altered immune responses, including central and peripheral inflammation in the disease progression, present evidence indicating the interaction between the central, and peripheral inflammation in both sporadic and familial PD, and discuss recent data supporting the key role of inflammatory responses in the initiation and progression of disease pathology.

## 2. Central Inflammation in PD

Microglia-associated central inflammation is a pathological hallmark of PD. Initial evidence of the involvement of inflammation in the progression of PD stems from a postmortem study over twenty years ago, which demonstrated the presence of activated microglia in the substantia nigra pars compacta (SNpc) of a PD patient [9]. Since then, an abundance of clinical and animal studies supports the role of activated microglia and increased levels of inflammatory mediators such as cytokines, chemokines, and ROS in the pathology of PD [10–14].

The central inflammatory process (activation/proliferation of microglia/astrocyte and secretion of proinflammatory cytokines and free radicals) could be an event secondary to the neurodegenerative process and, in turn, exacerbates the progression of cell death. However, under certain circumstances, inflammation could be a primary event that leads to the neurodegenerative process. A clinical study that uses positron emission tomography to study activated microglia in the brain of idiopathic PD patients shows the absence of significant longitudinal changes in microglia activation over time suggesting that brain inflammation occurs early in PD process [15]. We observe early inflammation in transgenic mice with overexpression or mutation (A53T and A30P) of *α*-Syn, characterized by microglial activation and increased expression in proinflammatory cytokines, prior to dopaminergic neuronal cell death and motor disorder [16, 17]. Another study using intranigral injection of lipopolysaccharide (LPS) on the dopaminergic system of the rat shows that LPS initially induces microglial activation in a short time (within 2 days) and subsequently causes a progressive degeneration of the dopaminergic system in the long term (up to one year after LPS injection) [18], suggesting that microglia-mediated inflammation underlies the neuronal cell death in the SN.

Activated microglia exert their neurotoxic effects by releasing proinflammatory cytokines such as TNF*α*, IL-1*β*, IL-6, and IFN*γ* and free radicals including ROS and NO, as well as inflammatory mediators such as prostaglandins E2 (PGE2), leading to nigral cell damage and death. Enhanced expression of TNF*α*, IL-1*β*, IL-6, and INF*γ* has been shown in basal ganglia as well as cerebrospinal fluid of PD patients [19–21]. TNF*α* and IL-1*β* are robust activators of NF-*κ*B and contribute to neuronal cell death by triggering apoptotic transduction pathway [22–24]. Additionally, TNF*α*, IL-1*β*, and INF*γ* induce potent activation of iNOS [25], presumably mediated by a low-affinity IgE receptor CD23, which is expressed exclusively on glial cells in the SN of PD patients [26]. iNOS is responsible for NO production, contributing to neuronal toxicity [27]. Collectively, cytokine/CD23-dependent activation of iNOS in microglia may be involved in the cascade of events leading to dopaminergic neuronal degeneration [28]. Moreover, TNF*α* and IL-1*β* can upregulate COX2, resulting in the production of PGE2 and induction of an intraneuronal toxic effect directly on dopaminergic neurons [29–31]. Given that dopaminergic neurons in the SN are relatively sensitive to “stress” [32] and that there is a large population of microglia in the SN in comparison to other CNS regions [33], inflammation is a crucial step in the pathogenic cascade leading to neurodegeneration.

Central inflammation pertains to all forms of PD, both genetic and idiopathic. Aging is a major factor for both genetic and idiopathic PD. Recent evidence suggests that aged brain resides in a chronic state of neuroinflammation, characterized by increased reactivity upon immune stimulation and low-grade production and central cytokines [18, 34–36]. One working hypothesis suggests that this hyperreactivity is due to priming of brain microglial cells. Microglia in aged brains become “primed,” exhibiting increased expression of MHC class II, scavenger receptor CD68 [37, 38], CD11b, and CD11c integrins [34, 39], Toll-like receptor TLR4 [40], and costimulatory molecule CD86 [41] and being capable of adopting a potent neurotoxic and proinflammatory phenotype [42]. Subsequent to peripheral innate immune stimulation, microglia in aged brains respond with an exaggerated inflammatory response compared to younger cohort [37, 43]. The precise priming stimulus of aging has yet to be identified. Some studies have suggested that IFN-*γ* concentrations are increased in the aged brain [44], which may implicate IFN-*γ* as a candidate molecule for microglial priming. To this end, the treatment of human microglial cell cultures with IFN-*γ* results in microglial activation, as evidenced by increased production of reactive oxygen species [45]. This scheme resembles the activation process of peripheral macrophages, wherein the classical macrophage activating factor, produced by stimulated Th1 lymphocytes and NK cells, is IFN-*γ* [46]. Other studies indicate that aging is associated with a reduction in anti-inflammatory cytokines including IL-10 and IL-4 [47, 48].

Astrocytes play equally important immunomodulatory role in maintaining CNS homeostasis. Altered astrocytic function is now recognized as a primary contributing factor to an increasing number of neurological diseases. With age, astrocytes have a more inflammatory profile. For instance, there is increased expression of astrocytic glial fibrillary acidic protein (GFAP) in the brain of aged rodents and humans [49, 50]. In addition, vimentin, an intermediate filament protein, also increases with aging in humans [51]. The age-related increases in GFAP and vimentin are similar to the activated astrocytic profile associated with inflammation and traumatic CNS injury [52]. There are many potential consequences of a more inflammatory astrocyte in the aged brain. First, astrocytes communicate directly with microglia, so an inflammatory astrocyte phenotype may elevate the amplitude and duration of microglia-mediated neuroinflammation [53]. Second, astrocytes play the most substantial role in maintaining an intact blood-brain barrier (BBB) [54]. Age-related changes in astrocytes can affect BBB permeability, especially under inflammatory conditions and neurodegenerative diseases [5, 55]. Third, astrocytes secrete chemokine monocyte chemoattractant protein-1 (MCP-1) which is a key chemokine involved in the recruitment of peripheral monocytes [56]. In summary, recent studies have shed some light on astrocyte-mediated neuroinflammation in neurodegeneration, and future research on astrocyte pathophysiology is expected to provide new perspectives on neurodegeneration and potential therapeutic strategies.

Several PD-linked genetic mutations are associated with increased glial activation in mediating chronic PD progression. A genetic dysfunction of *α*-Syn coupled with increased neuroinflammation can potentiate each other, driving chronic progression of neurodegeneration. Increasing experimental findings point to clear roles for *α*-Syn in the central inflammation in PD: (I) overexpression or mutation of *α*-Syn in the dopaminergic neurons leads to neuroinflammatory responses in *α*-Syn transgenic animals [16, 17]; (II) direct intranigral injection of *α*-Syn results in the production of proinflammatory cytokines and microglial activation in mouse brain [57]; (III) in the MPTP mouse model, nitrated form of *α*-Syn (N-*α*-Syn) is shown to drain into cervical lymph nodes and to elicit an antigen-specific T cell response. N-*α*-Syn specific effector T cells exacerbate microglial activation and DA neurodegeneration [58, 59]. Collectively *α*-Syn in SN DA neurons may elicit a self-propelling cycle of microglial activation and overproduction of inflammatory mediators in SN, leading to PD-associated dysfunction and spreading to neighboring neurons [60]. The mechanisms by which *α*-Syn initiates central inflammation remain to be determined. Recent data support the hypothesis that *α*-Syn is released from neurons into the interstitial space where the protein would be available to directly stimulate microglial activation via the scavenger receptor CD36 and the prostaglandin E2 receptor subtype 2 (EP2) [16, 17, 61, 62].

Mutations in Leucine-rich repeat kinase 2 (LRRK2) contribute to both idiopathic and familial forms of PD. LRRK2 expression is readily detected in multiple immune cells including B-lymphocytes, monocytes, dendritic cells, and microglial [63, 64], which suggests a potential role for LRRK2 in the immune system. PD-linked LRRK2 mutation (R1441G) increases proinflammatory cytokine release from activated primary microglial cells [65]. Furthermore, LRRK2 R1441G stabilizes cyclooxygenase 2 RNA and increases inflammatory response in idiopathic and genetic PD fibroblast [66]. In addition, LRRK2 in microglia plays a key role in the phagocytosis of neuronal elements [67]. In contrast, LRRK2 deficiency attenuates LPS-induced mRNA and/or protein expression of inducible nitric oxide synthase, TNF-*α*, IL-1*β*, and IL-6 [68]. Taken together, these results support LRRK2 as a positive regulator of inflammation in microglia, and disease-related LRRK2 mutations may shift the microenvironment of the brain to favor neuroinflammation.

Loss-of-function mutations in the E3 ligase Parkin give rise to a rare form or autosomal recessive parkinsonism [69]. Although mice deficient in Parkin do not display nigral degeneration, chronic administration of low-dose LPS trigger very similar neuroinflammatory and oxidative stress responses in the SNpc of both WT and Parkin-deficient mice; only the latter develops delayed and selective degeneration of DA neurons in SNpc but not in VTA [70]. These findings suggest that Parkin loss changes sensitivity to specific inflammatory mediators and increases vulnerability to inflammation-induced degeneration. Additional studies will need to establish whether and how Parkin-deficient glia is “primed” and respond aberrantly to exacerbate neurodegeneration.

The altered level or activity of certain gene products in CNS cells may contribute to central inflammatory response as well, although the mutation of these genes has not been identified in familial PD. For instance, the GPEX consortium reports a PD meta-analysis of gene expression data indicating that the mitochondrial master regulator, peroxisome proliferator-activated receptor gamma coactivator-1 alpha, PGC-1*α*, and related bioenergetic genes, including those encoding NADH ubiquinone oxidase (Complex I), succinate dehydrogenase (Complex II), cytochrome C oxidase (Complex IV), and ATP synthase (Complex V), are down-regulated in affected brain tissue from patients with both symptomatic and subclinical PD [71]. This observation suggests that PGC-1*α* is emerging as a molecular link between mitochondrial dysfunction and transcriptional dysregulation in PD.* In vivo* studies have shown that PGC-1*α* knockouts are much more sensitive to the neurodegenerative effects of MPTP, and increased PGC-1*α* levels protect neurons from oxidative stress* in vitro*, *α*-Syn-mediated cell death* in vitro*, and MPTP-mediated neuronal degeneration* in vivo* [72, 73]. Interestingly, a long-term study using muscle-specific PGC-1*α* knockout mice demonstrates that loss of muscle PGC-1*α* causes age-dependent low-grade, chronic inflammation in white adipose and liver tissue [74]. Whether PGC-1*α* deficiency in CNS cells will equally cause age-dependent low-grade inflammation in the brain remains unknown but warrant further investigation. Understanding the role of PGC-1*α* in the central as well as peripheral immune responses will provide new perspective for PD treatment.

## 3. Peripheral Inflammation in PD

The link between peripheral inflammation and neurodegeneration in PD patients has been revealed in several clinical reports. The clinical evidence for systemic inflammation in PD includes the presence of elevated serum levels of TNF*α* and TNF*α* receptor 1 in PD patients compared to control subjects [14, 75, 76]. Also, elevated plasma concentrations of IL-6 correlate with the increased risk of PD [77]. In addition, gut inflammation occurs in PD patients [78–80]. PD patients often suffer infectious disease, and the main causes of death are pneumonia and respiratory infections [81–84]. Furthermore, cytotoxic T lymphocyte (CD4+ and CD8+) has been described to infiltrate the SN of PD patients [85, 86]. The influx of these peripheral cells into the brain parenchyma could indicate a BBB dysfunction in PD patients [87, 88].

The association of peripheral inflammation and PD pathogenesis is further demonstrated in PD animal models. Pregnant rats exposed to intraperitoneal (i.p.) injection of LPS resulted in a decreased number of dopaminergic neurons in the pups when compared to nonexposed controls [89]. Similarly, rat fetuses exposed to LPS are more susceptible to 6-OHDA in adulthood [90, 91]. In adult animals, there is also data that strongly suggests the role of peripheral inflammation in the ongoing PD model. Animals with an increased peripheral inflammatory response after bacterial LPS injection are associated with central dopaminergic hypoactivity [92]. Peripheral inflammation induced by ulcerative colitis worsens the effects induced by intranigral LPS, including dopaminergic neuronal cell loss, microglial inflammation, and alteration in BBB permeability [93]. All previous data indicate a close relationship between the peripheral immune system and the central dopaminergic system.

It becomes more clear that peripheral inflammation plays a role in early stages of disease initiation and progression, including the development of preclinical nonmotor symptoms (hyposmia, constipation, bladder disorder, sleep disorder, obesity, and depression; Braak stages I and II [94]). Peripheral inflammation appears to accompany the nonmotor symptom of PD. One clinical study observes a significant correlation between serum TNF*α* levels and the severity of nonmotor symptoms including depression, sleep disturbances, and cognitive dysfunction in PD patients [95]. Gut infection and inflammation, mediated by a gram-negative bacterium,* Helicobacter pylori* (HP), are associated with PD [96, 97]. Moreover, successful treatment of HP has been shown to increase stride length in PD patients, whereas failure to eradicate HP results in significantly worse symptoms [98]. Chronic constipation, which occurs as early as 20 or more years before the onset of motor symptoms of PD, is casually linked to peripheral inflammation. Stomach infections may have early consequences on the enteric nervous system that manifest as gastrointestinal (GI) dysfunction including constipation [99]. Obesity, which is associated with increased risk of developing PD [100], displays increased levels of proinflammatory cytokines including TNF*α*, IL-1*β*, IL-6, and CCL2, in adipose tissue [101–103], liver [104], pancreas [105], brain [106], and possibly muscle [107]. Other studies have demonstrated that anti-inflammatory therapy may ameliorate MNS in non-PD related condition [108]. Collectively, strong clinical data support that peripheral inflammation appears to be an early event in the development of PD.

## 4. Interplay of Central and Peripheral Systems Drives Neurodgeneration

The CNS has been considered as immunologically privileged and protected by the BBB which prevents entry of pathogens and immune cells into the parenchyma. However, recent evidence suggests that the communication between central CNS and periphery is very fluid. BBB breakdown and systemic inflammation appear to play an important role in the pathology of numerous neurodegenerative diseases compromising the vascular unit and inducing leukocyte migration within the brain parenchyma [109]. A systemic infection or injury gives rise to an inflammatory response that communicates with brain. Both neural and humoral routes mediate communication from the site of peripheral inflammation to the brain. The neural route is through the dorsal motor nucleus of the vagus nerve [110]. The humoral route involves circulating cytokines that communicate with the brain via several routes: (i) by saturable transport across the BBB [111]; (ii) by activating endothelial cells and perivascular macrophages [112]; and (iii) through circumventricular organs which lack a functional BBB [113].

**Figure 1:**
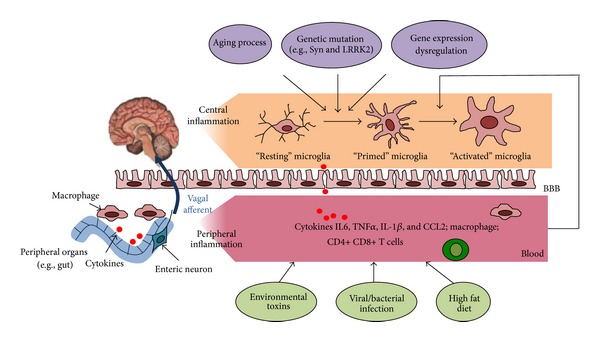
Interplay between central and peripheral immune systems in Parkinson's disease. The aging process, genetic mutation, and/or dysregulation of certain gene expressions serve as a “priming” stimulus for microglia. Upon secondary stimulation (e.g., environmental toxin, viral infection, high fat diet), peripheral inflammation is induced and communicates with brain through neural (vagal afferent) or humoral routs (e.g., cytokines circulation). The primed microglia are further activated and release excessive quantities of proinflammatory cytokines driving neurodegeneration.

Recent studies suggest that activation of the peripheral immune systems exacerbates the discordant central inflammatory response in aged or genetic predisposed brains. For instance, LPS challenge promotes microglial hyperactivity in aged mice, that is, associated with exaggerated induction of both proinflammatory IL1beta and anti-inflammatory IL-10 cytokines [114]. In addition, Gao et al. have established a two-hit animal model involving *α*-Syn mutation (A53T) and an environmental trigger (LPS) [115], which reproduces key features of PD and demonstrates synergistic effects of genetic predisposition and environmental exposures in the development of PD. Collectively, the aging process, genetic mutation, and/or dysregulation of certain gene expression serve as a “priming” stimulus for microglia, and upon secondary stimulation (e.g., environmental toxin or viral infection), the primed microglia release excessive quantities of proinflammatory cytokines driving neurodegeneration (Figure 1).

## 5. Therapeutic Implication

We are at the beginning of understanding the impact of chronic inflammation inside and outside of the brain towards neurodegenerative disorders. The more we are certain about these interactions, the better we would be able to diagnose, manage, and treat Parkinson's disease in a systemic but targeted manner.

Chronic inflammation in both sporadic and familial PD may represent therapeutic opportunities for immunomodulatory interventions in combination with other neuroprotective agents. However, the negative results of nonsteroidal anti-inflammatory drugs in late PD [116] strongly suggest that early immunomodulation is the key in preventing PD onset and progression. Minocycline, a broad-spectrum tetracycline antibiotic, has been tested in experimental models and PD patients. Minocycline effectively crosses the BBB and shows potent anti-inflammatory and neuroprotective effects in neurotoxin models of PD (e.g., MPTP and rotenone) [117]. A randomized, double-blind, Phase II futility clinical trial shows that minocycline offers clinical benefit to early PD patients, which warrants further consideration of minocycline for Phase III clinical trials [118]. Pioglitazone, a synthetic peroxisome proliferator-activated receptor gamma (PPAR-*γ*) agonist, is currently under investigation in a Phase II placebo-controlled clinical trial for the treatment of early PD.

LRRK2 is highly expressed in peripheral macrophage and monocytic cells as well as in central microglia, suggesting a functional role for LRRK2 in the innate immune system [119, 120]. Recent studies show that LRRK2 kinase inhibitors attenuate inflammatory signaling in HIV or LPS-treated microglia [67, 120]. Furthermore, small hairpin RNA targeting LRRK2 can equally inhibit LPS-induced microglial activation [120]. These data strongly suggest that inhibition or attenuation of LRRK2 is a promising therapeutic strategy for anti-inflammatory treatment for PD. However, it should be noted that LRRK2 knockout mice display alterations in exploratory and motor coordination behaviors and cause degeneration in the kidney [121], suggesting the wild-type LRRK2 may be involved in certain important normal physiological functions. Thus, a safe and effective LRRK2 therapeutic strategy using small molecule inhibitors or RNA interference should be specific and target disease-linked mutations.

PGC-1*α* is a potential new target for anti-inflammatory therapy for PD. Several pharmacological activators have been reported to enhance PGC-1a activity and stimulate mitochondrial biogenesis. PGC-1a activity is mainly controlled by the PPARs, AMPK, and Sirt1 [122]. Hence, pharmacological activators for these proteins have the potential to exert anti-inflammation as well as induce mitochondrial biogenesis through PGC-1*α* activation. Such activators include fibrates and rosiglitazone (PPAR) [123, 124], metformin [125], pyrroloquinoline quinone (PQQ) [126], and AICAR [127] (AMPK) as well as resveratrol (Sirt1) [128]. As PPAR agonist (fibrates, rosiglitazone) and AMPK activator (metformin, AICAR) are already routinely used in clinical practice for treatment of metabolic syndrome and Type 2 Diabetes, these drugs could be readily translated from animal models to PD patients. Preclinical CNS distribution and efficacy studies using inflammatory animal models of PD (i.e., the two-hit model) will be sufficient to warrant clinical trials on these drugs.

## 6. Conclusions

Although PD is complex multifactorial disorder with unknown etiology, increasing evidence supports an important role of central and peripheral inflammation in driving PD initiation and progression. Therefore, the current critical need is to identify promising targets for anti-inflammatory therapies, as well as fully understand the potential effects, both positive and negative, of blocking the inflammatory state in the early stage of the disease. Understanding the link between PD genetic variants or altered transcription and specific immune responses is crucial to identify novel therapeutic targets and to devise tailored neuroprotective interventions. As the research on preclinical/subclinical biomarkers for PD advances, anti-inflammation therapies clinical trials will become feasible for those at highest risk for PD.
